# Reorganization of the Neurobiology of Language After Sentence Overlearning

**DOI:** 10.1093/cercor/bhab354

**Published:** 2021-09-29

**Authors:** Jeremy I Skipper, Sarah Aliko, Stephen Brown, Yoon Ju Jo, Serena Lo, Emilia Molimpakis, Daniel R Lametti

**Affiliations:** Experimental Psychology, University College London, London, UK; Experimental Psychology, University College London, London, UK; London Interdisciplinary Biosciences Consortium, University College London, London, UK; Natural Sciences, University College London, London, UK; Experimental Psychology, University College London, London, UK; Speech and Language Sciences, University College London, London, UK; Wellcome Centre for Human Neuroimaging, University College London, London, UK; Experimental Psychology, University College London, London, UK; Department of Psychology, Acadia University, Nova Scotia, Canada

**Keywords:** brain, language learning, motor system, network organization, speech

## Abstract

It is assumed that there are a static set of “language regions” in the brain. Yet, language comprehension engages regions well beyond these, and patients regularly produce familiar “formulaic” expressions when language regions are severely damaged. These suggest that the neurobiology of language is not fixed but varies with experiences, like the extent of word sequence learning. We hypothesized that perceiving overlearned sentences is supported by speech production and not putative language regions. Participants underwent 2 sessions of behavioral testing and functional magnetic resonance imaging (fMRI). During the intervening 15 days, they repeated 2 sentences 30 times each, twice a day. In both fMRI sessions, they “passively” listened to those sentences, novel sentences, and produced sentences. Behaviorally, evidence for overlearning included a 2.1-s decrease in reaction times to predict the final word in overlearned sentences. This corresponded to the recruitment of sensorimotor regions involved in sentence production, inactivation of temporal and inferior frontal regions involved in novel sentence listening, and a 45% change in global network organization. Thus, there was a profound whole-brain reorganization following sentence overlearning, out of “language” and into sensorimotor regions. The latter are generally preserved in aphasia and Alzheimer’s disease, perhaps explaining residual abilities with formulaic expressions in both.

It is widely assumed that the brain regions supporting speech production, perception, and language comprehension are largely spatially fixed. Historical models consider “Broca’s area” the locus of speech production and “Wernicke’s area” the locus of comprehension (Tremblay and Dick 2016). Popular contemporary models still include these regions and a small number of others (anatomically correspond to aspects of the bilateral superior and middle temporal gyri, inferior parietal lobule, inferior frontal gyrus, and premotor regions) (Hickok and Poeppel 2007). Illustrating the fixity assumption, these are regularly described as “language regions” or “the language network,” with the latter phrase appearing in more than 6000 articles on Google Scholar (assessed June 2021).

Yet, there is a long history of evidence suggesting that language is more distributed throughout the brain than is belied by these models (Skipper 2015). This is illustrated by test–retest reliability studies that use language stimuli or that explicitly pertain to the reliability of regions involved in language processing. These show that stable individual participant activity patterns and networks are more variable and distributed than the set of aforementioned language regions (Burton et al. 2001). Though there are a number of reasons for this, a significant amount of the variance can be accounted for by individual differences in task strategies and cognitive style, like the tendency to visualize words (Miller et al. 2009, 2012).

A more specific example derives from the neurobiology of lexical processing. Words activate sensory and motor regions associated with their meaning, including language regions and far beyond. The written word “telephone” activates auditory cortex more than words that do not have auditory connotations (Kiefer et al. 2008), “red” activates the visual color region V4 (Martin et al. 1995), “kick” activates dorsal motor regions involved in moving the legs (Hauk et al. 2004), and “garlic” activates olfactory cortex (González et al. 2006). These activity patterns occur early, within 50–150 ms of word onset, while they are still being read or heard (Shtyrov et al. 2014). This implies that the increased involvement of sensory and motor regions is not simply a postperceptual process, somehow separable from true language regions.

These empirical examples suggest that the neurobiology of language is more distributed in the brain than popular contemporary models suggest. This difference might be explained by the reliance on measures of central tendency. That is, most experiments upon which these models are (presumably) based, average over individuals’ unique though reliable differences in language-related activity patterns. This results in language regions, even if the peaks of any given participant’s clusters are not in those regions (Burton et al. 2001). Similarly, most studies average over the differently distributed activity patterns associated with individual words from many unrelated semantic categories, leaving only language regions.

Individual differences and word processing are only 2 sources of spatial variability. Another potential source, related to both examples, is how well one has learned sequences of words. Neuroimaging studies using multiword or sentence stimuli typically average over word sequences that are more or less formulaic. “Formulaic expressions” are defined as being “prefabricated”, stored, and retrieved from memory as a whole and noncompositional (Wray and Perkins 2000). They are ubiquitous, comprising a third or more of everyday language (Conklin and Schmitt 2012), important for first and second language acquisition (Christiansen and Arnon 2017), and are processed faster and with fewer errors in both children and adults compared with novel words (Bannard and Matthews 2008; Arnon and Snider 2010).

The ability to produce formulaic speech is often preserved in aphasia, even with severe language impairment and damage to most or all of the language network (as in global aphasia) (Van Lancker Sidtis and Sidtis 2018). Where in the brain are formulaic expressions stored and processed when language regions are destroyed? Several not mutually exclusive possibilities exist. The first is a prominent theory that they are represented and processed by the right hemisphere and subcortical regions (Sidtis et al. 2018; Van Lancker Sidtis and Sidtis 2018). A more specific proposal is suggested by behavioral and corresponding cortical preservation in aphasia and Alzheimer’s disease. Lesion locations strongly associated with language do not typically include sensorimotor regions in or immediately around the central sulcus in either hemisphere, that is, primary motor and somatosensory regions (Dronkers et al. 2004; Baldo et al. 2013; Wilson and Schneck 2021). This is particularly true of comprehension but even mutism and nonfluent aphasia is more strongly associated with the left posterior inferior frontal gyrus than the central sulcus (Gorno-Tempini et al. 2006; Gunawardena et al. 2010; Montembeault et al. 2018; Wilson and Schneck 2021).

People with Alzheimer’s disease also produce more formulaic language than controls (Bridges and Van Lancker Sidtis 2013; Van Lancker Sidtis et al. 2015; Zimmerer et al. 2016) and the amount predicts disease progression (Zimmerer et al. 2016). As in aphasia, this phenotype corresponds to the relative degradation of temporal cortices and sparing of regions around the central sulcus (Thompson et al. 2003). In contrast, individuals with Parkinson’s disease produce less formulaic language (Van Lancker Sidtis et al. 2015) with a corresponding breakdown of cortical/subcortical sensorimotor networks (Sharman et al. 2013).

This work suggests that preserved formulaic language production in aphasia and Alzheimer’s disease is more reliant on sensorimotor regions and less on typical language regions, perhaps more in the right hemisphere. By extension, this implies that, as learning increases and words become more formulaic, production becomes more reliant on these same sensorimotor regions in healthy individuals. We take inspiration from this work in production and suggest that, by further extension, these same regions might also be involved in perceiving formulaic speech (Skipper et al. 2017). This is supported by neuroimaging studies of music and speech learning. These collectively show that perception after learning and consolidation involves more engagement of the production systems used during learning (Zatorre 2013). For example, in both monkeys and humans, learning to play sounds on a keyboard is subsequently associated with specific activation of sensorimotor regions involved in making finger and hand movements when listening to those sounds (Zatorre et al. 2007; Archakov et al. 2020).

What mechanistic account makes sense of “hearing” more with sensorimotor cortices and less with language regions? All listeners are faced with the problem of achieving perceptual constancy during speech perception. This is because there is variance in acoustic patterns both across and within talkers and no, as of yet, discoverable mapping between these patterns and speech categories (i.e., phonemes, syllables, etc.). We and others have argued that this difficulty might be solved if, during perception, the brain makes use of the contextual information that accompanies speech and the capacity of motor systems to predict the sensory consequences of movements (an ability important for motor learning and adapting in real time, known as “efference copy”) (Skipper et al. 2006, 2017; Skipper 2015).

For example, hearing “She was tired of her life and felt ready for a …” preactivates “change.” This is sequenced by sensorimotor regions involved in speech production as if it were to be spoken. Through efference copy, the motor pattern for producing “ch” activates acoustic patterns for “ch” in auditory cortices. If there is an overlap with incoming acoustic information, interpretation is confirmed, and further processing of change is unnecessary, conserving metabolic resources (relative to a less predictive context). Indeed, there is a large reduction in activity in the entire superior temporal plane in many predictive contexts during speech perception and language comprehension (Skipper 2014). By this account, the more overleared and formulaic a sequence of words, the earlier the whole sequence becomes predictable. In the example, change might be predictable at “ready” before overlearning but at “tired” after. Correspondingly, perception will be more supported by sensorimotor regions and much less by language regions.

Based on these speculations, we hypothesized that, as sentences become formulaic through production-based overlearning, there will be a reorganization of the brain regions supporting the perception of those sentences. Behaviorally, we operationalized overlearning as a decrease in reaction times to predict the final word of 2 sentences, to process the individual words in those sentences and the ability to accurately remember the sentences following 2 weeks of home production-based listening and repetition.

Neurobiologically, reorganization was predicted to correspond to a large increase in activity or new activations of sensorimotor regions. Here and throughout, we define “sensorimotor regions” as pre and primary motor and primary and secondary somatosensory cortices in and adjacent to the central sulcus. These and other regions were expected to support the production of novel sentences. Concomitantly, we predicted a large decrease or inactivity in language regions while listening to overlearned sentences. Language regions were defined as activation when listening to novel sentences not previously heard or overlearned. Finally, we expected these changes to correspond to a global network reorganization of the brain.

Importantly, we used a natural or so called “passive” design in which participants listened without making meta-linguistic judgments or corresponding button or vocal responses. This is because we hypothesized that sensorimotor regions involved in production support perception after overlearning. If we had included motor responses, there would be no verifiable way to argue that resulting sensorimotor activity was not due to those movements. Figure 1 provides an overview of the study design used to test these predictions.

**Figure 1:**
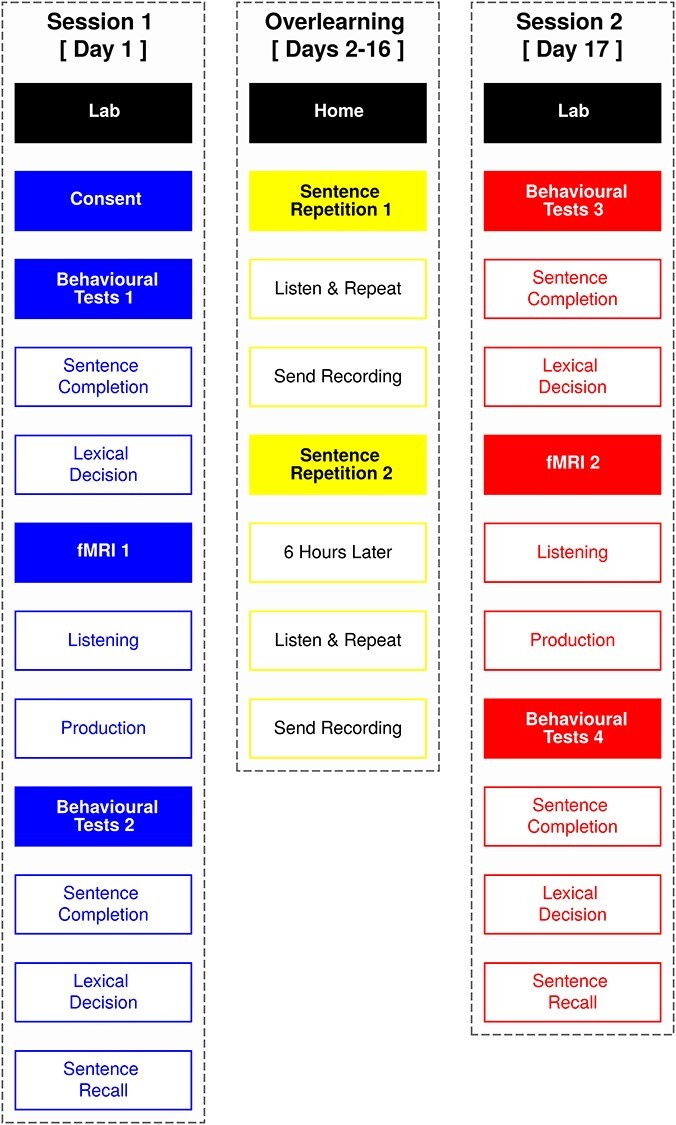
Study overview. We conducted fMRI over 2 sessions. In both sessions, participants passively listened to 2 sentences repeated 60 times each and 60 novel sentences. In a final run, participants produced the 60 novel sentences. The sessions were separated by 15 days. During this time period, participants trained at home by producing the 2 repeated sentences from fMRI 30 times each, twice a day. To assess learning, participants performed sentence completion, lexical decision, and sentence recall tasks before and after training.

## Materials and Methods

### Participants

There were 12 participants (6 females; 21–25 years old; M = 23.17; standard deviation [SD] = 1.41). All were native speakers, with British English being the only language spoken at home before the age of 5. They could speak other languages though all but one self-reported being “monolingual” in that they did not speak any other language fluently. Participants were right-handed as determined by the Edinburgh Handedness Inventory (Oldfield 1971). All had unimpaired hearing and (corrected) vision. None had any contraindication for magnetic resonance imaging (MRI), history of psychiatric or neurological disorder, or language-related learning disabilities. All participants gave informed consent, and the study was approved by the University College London Ethics Committee.

### Procedure

The experiment lasted 17 days, including 2 testing days, each with 3 different behavioral tasks (completed multiple times) and functional magnetic resonance imaging (fMRI) to assess overlearning of 2 sentences (Fig. 1). On the first day, participants performed a sentence completion and lexical decision task on a desktop computer in a noise-attenuated testing room, using headphones. Both tasks included the words from the 2 sentences that the participants would overlearn over the subsequent 15 days.

Following these tasks, participants were escorted to the scanning suite. There they chose comfortable earbud sizes for noise-attenuating headphones. After being instructed, they were put in the head-coil with pillows under and on the sides of their head and (if desired) under the knees for comfort and to reduce movement during scanning. Once in place, participants chose an optimal stimulus volume by determining a level that was loud but comfortable. Once scanning began, participants’ first fMRI task was to “passively” listen to the 2 “overlearned” sentences multiple times and previously unheard or “novel” sentences. Participants’ last fMRI task was to listen to and repeat some of the novel sentences that they had earlier heard in the scanner. Finally, we acquired high-resolution anatomical scans. After scanning, participants were returned to the testing room where they did the sentence completion and lexical decision tasks a second time and a sentence recall task to assess learning over the first day.

Over the next 15 days, participants trained at home by listening to and repeating the 2 overlearned sentences they heard during fMRI. They did this twice a day, sending us recordings of their productions when they were done. Participants returned on the final (17th) day and performed the sentence completion, lexical decision and sentence recall behavioral tasks, fMRI passive listening and speech production tasks, and anatomical scans as on day 1, in the same order. When these were complete, participants were given £7.5 per hour for behavioral testing and £10 per hour for scanning to compensate for their time and sent home.

### Stimuli

Participants were divided into 3 groups. Each group of 4 participants overlearned 2 different sentences. Three pairs of sentences were used to help assure that results are generalizable. Specifically, a male talker recorded the 498 sentences from the supplemental materials of Block and Baldwin (2010). Sentences were edited in Praat (http://www.fon.hum.uva.nl/praat/) to be 2.5 s, with the final word lasting 500 ms, with the latter being approximately the average length of a spoken word (Tucker et al. 2019). This was done to assure that the timing was the same for all sentences so that differences in fMRI activation patterns could not be attributed to subtle differences in the length of stimuli (see next “Sentence Completion” section for a further rationale). Four raters judged whether the sentences were appropriate for British English listeners (e.g., they do not discuss American football or “pants”) and sounded natural (i.e., they were not sped up or slowed down). The latter was done using a Likert scale from 1 (not natural) to 10 (very natural). Inappropriate sentences, those with a naturalness rating less than 6 and sentences with proper nouns were discarded. From the remaining sentences, 3 sets of 2 sentences were created that were matched on number of words, cloze probability (i.e., the probability of the final word completing the preceding words), complexity, and average word frequency as determined by Subtlex-UK (Van Heuven et al. 2014) (Table 1). From the remaining sentences, 60 high cloze probability sentences were selected to be used in behavioral tasks and during scanning.

**Table 1:** Overlearned sentence information

Set	Sentence	Words	Cloze probability	Complexity	Average frequency
1	Instead of dressing, I prefer vinegar and oil.	8	0.76	1.2	7716.87
	At the pub, he ordered another mug of beer.	9	0.87	1.2	8645.02
2	She was tired of her life and felt ready for a change.	12	0.83	1.3	7134.33
	The jury found him innocent and set him free.	9	0.85	1.3	7996.94
3	They went to the video store to rent a movie.	10	0.54	1.4	13093.97
	She followed the recipe correctly to cook the meal.	9	0.56	1.4	13651.17

All sentences were 2.5 s long and the final word was 500 ms.

### Behavioral Tasks

Three behavioral tasks were used to assess overlearning of the 2 sentences assigned to each participant. Sentence completion and lexical decision tasks were completed both before and after the 2 fMRI sessions. The sentence recall task was done only after each imaging session.

### Sentence Completion

Participants listened to 2 sentences that were to be overlearned (sessions 1 and 2) or had been overlearned (sessions 3 and 4) and 30 novel sentences. In each case, the final word in the sentence was removed. Participants were asked to press a button as soon as they believed they knew the final word. The sentence stopped playing when the button was pressed and participants then typed in the final word as quickly as possible. Reaction times for the task were measured from the start of sentence playback until participants finished entering the predicted final word. Because all of the sentence frames were the same length, a fixed and objective measure of reaction time changes could be determined (i.e., from 0 to 2000 ms). On a per participant basis, reaction times greater than 4 SDs from the mean reaction were not included in the analysis.

#### Lexical Decision

Participants had to judge the lexical status (word or not word) of overlearned words, words similar to overlearned words, words dissimilar to overlearned words, and nonwords. Thirty overlearned words were extracted from the overlearned sentences used in the study. Eighteen similar words were selected by searching through the remaining novel sentences and finding words that had a Levenshtein distance of 2 or less from the overlearned words extracted from the overlearned sentences. Levenshtein distance is the minimum number of single character alterations required to change one word into another (Levenshtein 1966). Sixteen dissimilar words were also identified. These had a Levenshtein distance of 6 or more and also had a frequency that differed by ±10% from the overlearned words. Sixty-four unintelligible but speech-like nonwords were created from the selected (i.e., overlearned, similar, dissimilar) words using a local time reversal script in Praat with 150-ms steps (Saberi and Perrott 1999).

Each participant heard only 21 words (9 overlearned words taken from the 2 sentences they repeated, 6 similar words, and 6 dissimilar words) and 21 nonwords (reversed versions of the overlearned, similar, and dissimilar words they heard). After hearing each word they indicated if they heard a word or a nonword with a button press as quickly and accurately as possible. Reaction times were measured from the start of playback to the moment a response was indicated. On a per participant basis, reaction times greater than 4 SDs from the mean reaction were not included in the analysis. We then examined how reaction times changed between the 2 scanning sessions.

#### Sentence Recall

After scanning sessions, participants were asked to type in up to 10 sentences that they remembered hearing in the scanner. The sentences they recalled (e.g., “He was a lousy cook and ordered out”) were matched (based on semantics and word use) to the sentences that were actually played in the scanner (e.g., “He eats out because he is a lousy cook”). The cosine similarity was then found between the 2 sentences to provide a measure of recall accuracy. The cosine similarity is the cosine of the angle between 2 normalized vectors. In this case, the 2 sentences were transformed into vectors of word counts and the cosine similarity was found for these word count vectors. Cosine similarity takes a value between zero and one, where a value of zero means that the sentences share no words and a value of one means that the sentences are identical in terms of words used.

#### Sentence Overlearning

Commencing the day after the first fMRI session, participants overlearned 2 sentences through repetition at home, twice a day. Each day, participants listened to a prerecorded set of their 2 sentences, each repeated 30 times in a random order, lasting 5 min and 32 s. There was a 3-s gap between sentences during which the participant repeated the sentence out loud. The participants did this task again at least 6 h later using another randomized prerecorded file of the same 2 sentences. All 30 prerecorded files had a different randomization. To verify home learning took place, participants recorded themselves listening to and repeating their sentences using Audacity (http://www.audacityteam.org) and immediately shared the recording to a cloud storage folder. In total, participants listened and repeated their 2 sentences 1800 times for 2 h and 46 min. This was verified by checking recordings.

### fMRI

#### Task

A slow random event-related design was used to compare how the response to overlearned sentences changed from fMRI session 1 to 2. In each scanning session, audio stimuli were presented during 6 listening runs and a speech production run. Each listening run lasted 6 min and 53 s. Across these, participants heard 2 sentences repeated 60 times each and 60 novel sentences that were each only heard once, all in a randomized order. Stimuli were presented in a jittered manner such that, following each 2.5-s sentence, there was a minimum of 10 s of silence and a mean of 10.675 s (SD = 0.93) and a maximum of 15 s of silence. The speech production run always followed the 6 listening runs and lasted 8 min and 10 s. In this run, participants listened to 30 of the novel sentences they had just heard and were asked to repeat each sentence as soon as it finished. After each 2.5-s sentence and allowing for another 2.5-s period to produce the sentence, there was, again, a minimum of 10 s of silence with a mean of 10.69 s (SD = 1.34) and a maximum of 15.625 s of silence. All listening and production runs included 10 s of silence at the start to allow magnetization to reach a steady state. Sessions 1 and 2 were the same, with the exception that in session 2 they produced the other 30 sentences not produced in session 1. Participant engagement was monitored with a camera over 1 eye.

Functional and anatomical images were acquired on a 1.5 T Siemens MAGNETOM Avanto with a 32 channel head coil (Siemens Healthcare, Erlangen, Germany). We used multiband echo-planar imaging (EPI) (Feinberg et al. 2010; Feinberg and Setsompop 2013) (time repetition [TR] = 700 ms, time echo [TE] = 54.8 ms, flip angle of 75°, 28 slices, resolution = 3 × 3 × 4 mm), with ×4 multiband factor and no in-plane acceleration. Slices were manually obliqued to include the entire brain. The 6 listening EPI runs were each 590 volumes/TRs and the speech production run had 700 volumes/TRs. Two 6-min *T*_1_-weighted high-resolution MPRAGE anatomical MRI scans followed the functional scans (TR = 2.73 s, TE = 3.57 ms, 176 sagittal slices, resolution = 1.0 mm^3^). Imaging parameters were the same for both fMRI sessions (thus resulting in 12 listening, 2 production runs, and 4 anatomical scans).

#### Preprocessing

Unless otherwise noted, the AFNI software suite was used for preprocessing and analyses (http://afni.nimh.nih.gov/afni) (Cox 1996). Individual AFNI programs are indicated parenthetically in subsequent descriptions.

The 4 anatomical/structural MRI scans were corrected for image intensity nonuniformity (“3dUniformize”) and deskulled using “ROBEX” (Iglesias et al. 2011). Within each session, the second anatomical image was aligned to the first and they were averaged. Then the resulting sessions 1 and 2 anatomical images were aligned and averaged. This was done using a procedure to reduce bias by moving both anatomical images, so that both are interpolated some amount rather than 1 session receiving all the interpolation (https://sscc.nimh.nih.gov/sscc/dglen/alignmentacross2sessions).

The resulting anatomical image was nonlinearly aligned (using “auto_warp.py”) to the MNI N27 template brain, an average of 27 anatomical scans from a single participant (“Colin”) (Holmes et al. 1998). The anatomical scan was inflated and registered with Freesurfer software using “recon-all” and default parameters (version 6.0, http://www.freesurfer.net) (Fischl 2012). This included automatic parcellations of the anatomical image. These were used to create white matter and ventricle (i.e., cerebral spinal fluid containing) regions of interest that were used as noise regressors. Automatic parcellation was also used to generate 167 regions of interest for network analyses (i.e., using the “Destrieux Atlas”) (Destrieux et al. 2010).

The first 10 TRs (7 s) were removed from the fMRI time series before they were corrected for slice-timing differences (“3dTshift”) and despiked (“3dDespike”). Next, volume registration was done by aligning each timepoint to the mean of run 4 (“3dvolreg”). The functional data were then aligned to the anatomical images (“align_epi_anat.py”). This used the less biased procedure described for anatomical alignment, moving functional data from both sessions so that no one session received all the interpolation. Finally, the volume-registered and anatomically aligned functional data were (nonlinearly) aligned to the MNI template brain (“3dNwarpApply”).

Next, we created 2 sets of time series. The first, to be used in the deconvolution analysis described below, involved only normalizing each run to have a sum of squares of one (“3dTproject”). The second set of time series were normalized and detrended using Legendre polynomials whose degree varied with run lengths (following the AFNI recommended formula of [number of timepoints * TR]/150). These were then submitted to spatial independent component analysis (ICA) to detect and remove artifacts. This was done because we did not collect physiological data, used a multiband sequence, and had a speech production task, all considered to be sources of noise amenable to correction by ICA (Griffanti et al. 2017). Specifically, we concatenated the normalized and detrended listening and production time series from both sessions separately (“3dTcat”). We did ICA on the resulting listening time series with 300 dimensions and on the production time series with 100 dimensions using “melodic” (version 3.14) from FSL (Smith et al. 2013). Next, we labeled and removed artifacts from the time series, following recommendations from an existing guide for manual classification (Griffanti et al. 2017). One of 2 trained authors went through all components and associated timecourses, labelling the components as “good,” “maybe,” or “artifact.” Our strategy was to preserve signal by not removing components classified as “maybe.” Using this approach, 78.75% of the listening and 77.16% of the resulting production components were labeled as artifacts, comparable to prior work with ranges between 70% and 90% (Griffanti et al. 2017; Aliko et al. 2020).

Finally, we made a third time series using the concatenated listening runs for the regional homogeneity analysis described below. Specifically, the time series were normalized to have a sum of squares of one and detrended (“3dTproject”) with the following regressors: 1) Legendre polynomials whose degree varied with run lengths (following the previously described formula); 2) 6 demeaned motion regressors from the volume registration (roll, pitch, yaw, and changes in the inferior/superior, left/right, and anterior/posterior directions); 3) a demeaned white matter activity regressor from the averaged time series in white matter regions; 4) a demeaned cerebrospinal fluid regressor from the averaged time series activity in ventricular regions; and 5) the ICA artifact component timecources.

#### Individual Deconvolutions

After preprocessing, 2 individual participant deconvolutions were conducted to get an estimation of the system impulse response function for the 1) overlearned and novel sentences from the listening runs and the 2) produced novel sentences from production run from both fMRI sessions (“3dDeconvolve”) (Glover 1999). In the first deconvolution, regressors of interest included 1 each for overlearned sentence 1 in session 1, overlearned sentence 2 in session 1, novel sentences in session 1, overlearned sentence 1 in session 2, overlearned sentence 2 in session 2, and novel sentences in session 2. For each of these, the hemodynamic response was estimated using a cubic spline basis function that covered an 18-s period after each stimulus onset, using 20 tent functions to generate the impulse response function for every voxel. In a second deconvolution, regressors of interest included speech production of novel sentences in session 1 and speech production of novel sentences in session 2. Again, the cubic spline basis function was used with the difference that the period covered was 20 s (to account for the extra time involved in producing the sentences), using 21 tent functions.

In both deconvolutions, regressors of noninterest included an automatically estimated number of polynomials (again following the [number of timepoints * TR]/150 formula), 6 motions regressors from the volume registration step (roll, pitch, yaw, and changes in the inferior/superior, left/right, and anterior/posterior directions), 2 regressors from the average time series in the white matter and ventricles, and all timecourses from ICA components labeled as artifacts.

#### Novel LMM

Following individual participant deconvolutions, the resulting impulse response functions were spatially smoothed to achieve a level of smoothness of 6-mm full-width half maximum, regardless of the smoothness it had on input (“3dBlurToFWHM”) (Friedman et al. 2006). These were then used in 4 linear mixed-effects models (LMMs; “3dLME”) (Chen et al. 2013).

First, we did a novel sentence listening LMM. Factors were session (1 and 2) and timepoint (0–25). This allowed us to test the prediction that learning was specific to overlearned sentences. Though we did not expect differences in activity for the novel sentences across sessions, participants might have learned something about the talker’s voice as the same talker made all sentences used in the study. Time was included as a factor to compare to the overlearning LMM (see next paragraph) though, again, we did not expect that there would be differences in the shapes of the hemodynamic response between sessions 1 and 2. There were 26 timepoints because our TR was 700 ms and there are, therefore, 26 TRs covering the 18-s period from the deconvolution. Finally, collapsing over session and time, the novel LMM served to identify language regions that were expected to encompass the bilateral inferior frontal gyrus and superior temporal plane.

#### Overlearning LMM

Second, we conducted an overlearning sentence listening LMM with sentence (overlearned sentence 1 and 2), session (1 and 2), and timepoint (0–25) as factors. This allowed us to understand the effect of learning on overlearned sentence listening between sessions 1 and 2. Time is included as a factor because we expect differences in the shapes of the hemodynamic response across sessions though we did not make a priori predictions about the direction of those differences in individual brain regions. We also visualized the timepoints from the results to better understand if responses for overlearned sentences are a simple redistribution of activity (i.e., a relative modulation of activity from sessions 1 to 2) or a reorganization of brain responses (i.e., activity in regions in session 1 or 2 that was not previously present).

#### Overlearning−Novel LMM

Third, a follow-up analysis was conducted by subtracting the novel from the overlearned impulse response functions at each timepoint in each participant. We then ran a LMM with the same factors as the overlearning sentence listening LMM (i.e., session*sentence*timepoint). This allowed us to formally test whether overlearned sentences produced significantly more activity than novel sentences in sensorimotor regions and less activity than novel sentences in language regions. We present the results of each session separately so that the direction of effect can be interpreted.

#### Production LMM

Finally, we did a novel speech production LMM with session (1 and 2) and timepoint (0–28) as factors. This analysis was used to demonstrate regions involved in producing the novel sentences that participants heard during both sessions.

#### Multiple Comparisons Corrections

To correct for multiple comparisons in all LMMs, we used a multi-threshold approach rather than choosing an arbitrary *P* value at the individual voxel level as is customary. In particular, we used a cluster simulation method to estimate the probability of noise-only clusters using the spatial autocorrelation function from the residuals in each LMM (“3dFWHMx” and “3dClustSim”). This resulted in the cluster sizes to achieve a corrected alpha value of 0.01 at 9 different *P* values (i.e., 0.05, 0.02, 0.01, 0.005, 0.002, 0.001, 0.0005, 0.0002, and 0.0001). We thresholded each map at the corresponding z-value for each of these 9 *P* values and associated cluster size. We then combined the resulting maps, leaving each voxel with its original z-value.

#### Regional Homogeneity

The described deconvolution approach uses a linear model to derive an estimate of the hemodynamic response from multiple stimulus presentations. We reasoned that a strong case for the hypothesis could be made if a similar set of results were obtained using a more model-free approach across the whole time series. To do this, we used regional homogeneity that calculates the Kendall’s coefficient of a concordance for each voxel within a neighborhood of voxels (“3dReHo”). We chose this particular approach because it is also a measure of local interactions, synchronization, and connectivity (Jiang and Zuo 2016). Corresponding to our hypothesis, we expected an increase in local connectivity in sensorimotor regions and a decrease in superior temporal plane and inferior frontal regions after overlearning.

To do this analysis, we first constructed 3 time series that theoretically reflect only timepoints for processing overlearned sentences from session 1, overlearned sentences from session 2, or novel sentences from both sessions. Specifically, we modeled expected hemodynamic responses by convolving stimulus onsets with a canonical hemodynamic response function (using “WAV”, a.k.a the “Cox special” from “waver”). We then cut up the third time series described in “Preprocessing” above by taking the relevant timepoints under the canonical response starting from the timepoint that the response starts to rise (a delay of 2.1 s or 3 TRs) and ending when the response returns to baseline (“3dTcat”). We removed any timepoints that overlapped in any of the time series. Because this was a slow event-related design with jitter, this amounted to only 7.02% of the data that was about equally distributed across the 3 sentence time series.

We then did the regional homogeneity analysis for each of the 3 resulting time series using a radius of 2.3, which equals 57 voxels (we also tested 2.0 or 33 voxels and 2.9 or 93 voxels and it makes little difference to the results). The resulting maps were then blurred to achieve a level of smoothness of 6-mm full-width half maximum (“3dBlurToFWHM”). After this, we conducted 3 group paired *t*-tests to compare 1) the overlearned sentences from session 1 to those from session 2; 2) the novel sentences from sessions 1 and 2 to the overlearned sentences from session 1; and 3) the novel sentences from sessions 1 and 2 to the overlearned sentences from session 2 (“3dttest++”).

We saved the results as *z*-scores and used the residuals from the *t*-tests to do the same multi-thresholding procedure described for the LMM analyses to correct for multiple comparisons. We note that “3dttest++” has a built-in and more sophisticated equitable thresholding procedure (called equitable thresholding and clustering (ETAC)) (Cox 2019). However, at the time of analysis, there was no obvious way to use this approach with, for example, the LMM results. As such, we opted to use our described approach for consistency across analysis. That said, we looked at the results using the ETAC approach for comparison and they look similar to our multi-thresholding approach.

#### Network

To do network analyses, we first concatenated the 2 overlearned sentence impulse response functions from the deconvolution for the 2 sessions separately. We blurred these as in previous analyses to a level of smoothness of 6-mm full-width half maximum (“3dBlurToFWHM”). Using the Freesurfer “Destrieux Atlas” parcellation, 167 regions of interest were extracted from these time series for each participant. Pairwise Pearson’s correlations were used to build 2 unweighted, undirected adjacency matrices for each participant, 1 each for sessions 1 and 2 overlearned sentences. Absolute thresholding of *r* = 0.1 was applied to correlation values, in order to build adjacency matrices for group comparisons (Garrison et al. 2015).

To test whether global network reorganization took place after overlearning, the distance measures “edit distance” and “Deltacon” were calculated. Edit distance computes additions or deletions of connections between 2 graphs (Wills and Meyer 2020). The edit distance matrix was defined as:

$$ \delta \left(G,G^{\prime}\right)=\left\Vert\ A-A^{\prime}\right\Vert $$

Where A and A’ are the adjacency matrices for graphs G (session 1) and G’ (session 2), respectively, and δ is the pairwise edit distance (Wills and Meyer 2020). Since session 1 and 2 shared node identity, this pairwise application was applicable. Change in connectivity was calculated as the ratio between the total number of lost and gained connections in the edit distance matrix and the total number of connections in both sessions 1 and 2 adjacency matrices for a single participant. A 1-sample *t*-test across participants was performed on the change in connectivity values to determine if they differed from the null, that is, no change in connectivity.

In order to describe and visualize connections involved in edit distance differences from sessions 1 to 2, a 2-way chi-square test was performed on each region of interest pair across participants. Lost connections were defined as those whose connectivity in session 2 was lower, whereas observed connectivity in session 1 was higher than expected. Gained connections were defined as the converse. We set a threshold at *P* < 0.01 to afford some protection for multiple comparisons.

Though the edit distance matrix is simple to compute, it suffers from limitations. It only determines specific connection changes, but it does not interpret the change in the context of the rest of the network and its neighbors. Moreover, it does not differentiate between network densities: If a connection is lost in a very sparse network the result would be a large disruption, but if a connection is lost in a highly dense network the outcome on the global scale will be minimal (Koutra et al. 2013). For these reasons we also calculated Deltacon, a more robust similarity measure that determines the level of isomorphism between 2 networks with node correspondence, using Matusita’s distance (Koutra et al. 2013). We compared the results using a 1-sample *t*-test across participants on the deltacon dissimilarity value (i.e., 1-Deltacon).

To further examine possible changes between sessions 1 and 2, we explored a number of other global network measures. These included density, diffusion, efficiency, and flow using the Brain Connectivity Toolbox in MATLAB (Rubinov and Sporns 2010). Density measures how “connected” a network is, diffusion how quickly information can get from point A to B, efficacy the average inverse shortest path length (Ek et al. 2015), and flow how centralized a network is for transfer of information (Rubinov and Sporns 2010). We used a 2-sample *t*-tests for these global measures, comparing sessions 1 and 2. Finally, we computed 2 measures of local connectivity, centrality, and community partitioning. Centrality (degree, eigenvector, closeness, and betweenness) measures the importance of a node in a network, whereas community detection partitions the network into distinct subcomponents or modules (Rubinov and Sporns 2010).

## Results

### Behavioral Tasks

**Figure 2:**
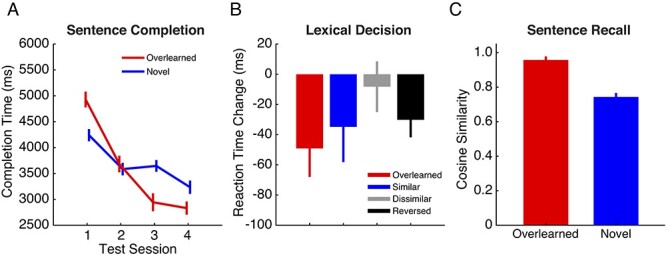
Behavioral measures of overlearning. (*A*) For each testing session (1–4), sentence completion is the length of time (in ms) that it took participants to type in the final word of overlearned (red line) and novel sentences (blue line). Completion times decreased following overlearning. (*B*) Lexical decisions (word/not word) were made for words drawn from the overlearned sentences (red bar), similar words (blue bar), dissimilar words (gray bar), and reversed words (black bar). Change in lexical decision time reflects decision times (in ms) measured in sessions 1 and 2 (preoverlearning) versus decision times measured in sessions 3 and 4 (postoverlearning). (*C*) Participants recalled sentences they heard during scanning. The accuracy of sentence recall was measured as the cosine similarity between recalled and heard sentences. Participants were more likely to recall sentences they had overlearned (red bar) versus novel sentences (blue bar).

We hypothesized that participants would show behavioral markers of overlearning for the sentences repeated at home. We assessed this with 3 behavioral tasks (Fig. 2).

#### Sentence Completion

Figure 2*A* shows sentence completion times (in which participants completed the final word of a sentence) across testing sessions for both overlearned and novel sentences. There was an interaction between completion time measured at each session and whether the sentence was overlearned or not (*F*_3,33_ = 22.28, *P* < 0.001). For overlearned sentences, a decrease in sentence completion times was observed following training (session 2 versus session 3; *t*_11_ = 3.90, *P* < 0.01, Cohen’s d = 1.12). A similar decrease was not observed for novel sentences (*t*_11_= −1.04, *P* = 0.32, Cohen’s d = 0.29). By the end of the study, participants completed the final word in the overlearned sentences 2.10 s faster than they did at the start of the experiment (mean reaction time (RT) in session 1 = 4.94 s vs. mean RT in session 4 = 2.84 s). Instead, the time it took to complete the final word in novel sentences improved by 1.01 s (mean RT in session 1 = 4.25 s vs. mean RT in session 4 = 3.24 s).

#### Lexical Decision

Figure 2*B* shows changes in lexical decision times following overlearning (testing sessions 1 and 2 vs. sessions 3 and 4), for words drawn from overlearned sentences (red bar), words similar to overlearned words (blue bar), words dissimilar to overlearned words (gray bar), and nonwords (black bar). Changes in lexical decision time between the 4 word types were not significantly different (*F*_3,33_ = 2.32, *P* = 0.094). However, words drawn from overlearned sentences were identified faster following overlearning than they were before training (*t*_11_ = −2.59, *P* = 0.025, Cohen’s d = −0.75). The mean change in RT for words drawn from overlearned sentences was −49.13 ms. A comparable result was not observed in the cases of similar (*t*_11_ = −1.49, *P* = 0.16, Cohen’s d = −0.43) and dissimilar words (*t*_11_ = −.49, *P* = 0.63, Cohen’s d = −0.14). The mean change in RT for words drawn from similar and dissimilar sentences were −33.88 and −8.25 ms, respectively.

#### Sentence Recall

Figure 2*C* gives a measure of sentence recall (i.e., memory) for overlearned sentences versus novel sentences following overlearning. Participants recalled overlearned sentences with significantly greater accuracy than novel sentences (*t*_11_ = 8.00, *P* < 0.001, Cohen’s d = 2.37). The cosine similarity between the overlearned sentences and what was recalled averaged 0.96, whereas the cosine similarity between the novel sentences and what was recalled averaged 0.74. Indeed, half of the participants recalled the overlearned sentences verbatim. By comparison, none of the participants recalled the novel sentences verbatim even though they had just heard them during scanning. Nonetheless, novel sentence recall was high enough to suggest that participants paid attention during scanning.

### fMRI

#### Novel LMM

**Figure 3:**
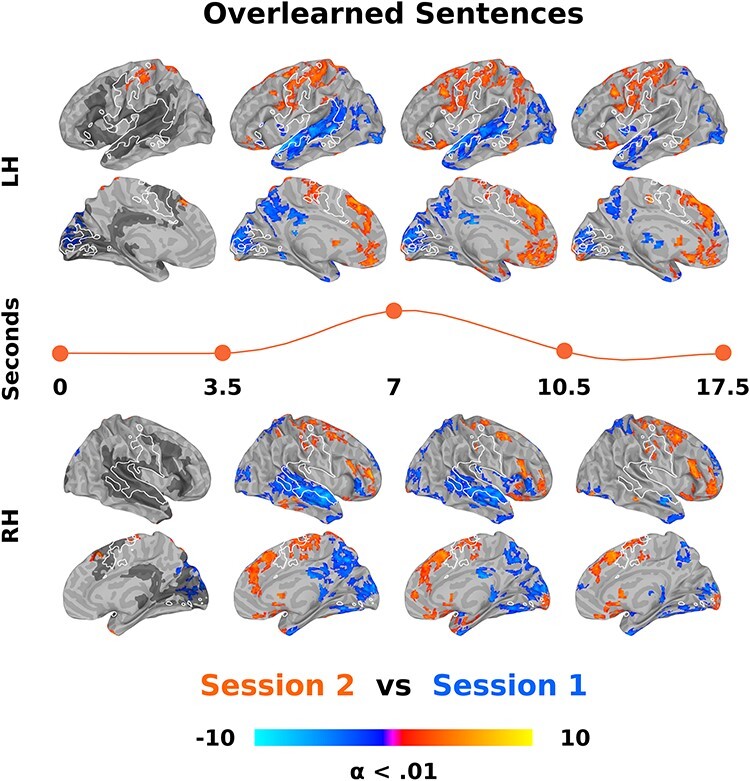
Direct contrast of overlearned sentence listening from sessions 1 to 2 across time. GLTs between sessions 1 and 2 overlearned sentences were done at each of 26 timepoints in the estimated impulse response functions following an LMM. For ease of visualization, these contrasts are collapsed into 4 time bins between the indicated seconds. The orange line separating the left (LH) and right (RH) hemisphere surfaces roughly represents the relative position in a “canonical” hemodynamic response function. Listening to sentences after overlearning results in an increase in activity in sensorimotor regions centered around the central sulcus (among other regions, reds) and a reduction of activity in the superior temporal plane (blues). Increased sensorimotor activity overlaps with producing novel sentences as determined by a separate LMM (white outline). Note that the sensorimotor regions are first engaged after overlearning in the delay period before the canonical response rises. Decreased superior temporal plane activity overlaps with listening to novel sentences as determined by another LMM (dark gray, presented on only the far left column though it represents all 26 timepoints). Each timepoint was cluster size corrected for multiple comparisons at alpha (α) < 0.01. The color bar represents *z*-scores. See also Table 2.

**Figure 4:**
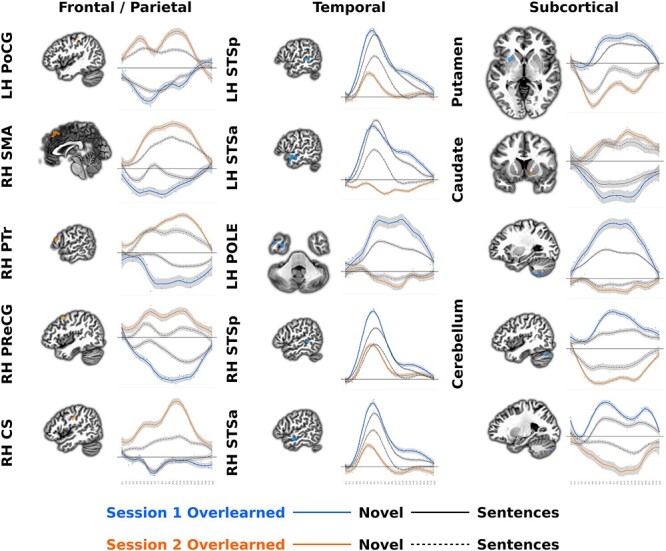
Estimated impulse response functions for overlearned and novel sentence listening in different regions. The direct contrast of overlearned sentences from sessions 1 to 2 was thresholded at *z* = 10 (*P* < 1.52 × 10^−23^), resulting in 40 clusters of activity. Fifteen of these, 5 each from frontal/parietal, temporal, and subcortical regions were selected for illustrative purposes (Fig. S4 for the other 25 clusters). The impulse response function from the voxels in each of these clusters from the overlearning LMM were averaged and plotted for each session. Also plotted are the averaged novel sentence listening LMM impulse response functions for comparison. Note that in most cases, the changes in the impulse response functions before or after learning are from a state of below baseline or inactivity. Abbreviations: CS = central sulcus; PoCG = postcentral gyrus; POLE = temporal pole; PreCG = precentral gyrus; PTr = pars triangularis of the inferior frontal gyrus; SMA = supplementary motor area; STSa = anterior superior temporal sulcus; and STSp = posterior superior temporal sulcus.

**Figure 5:**
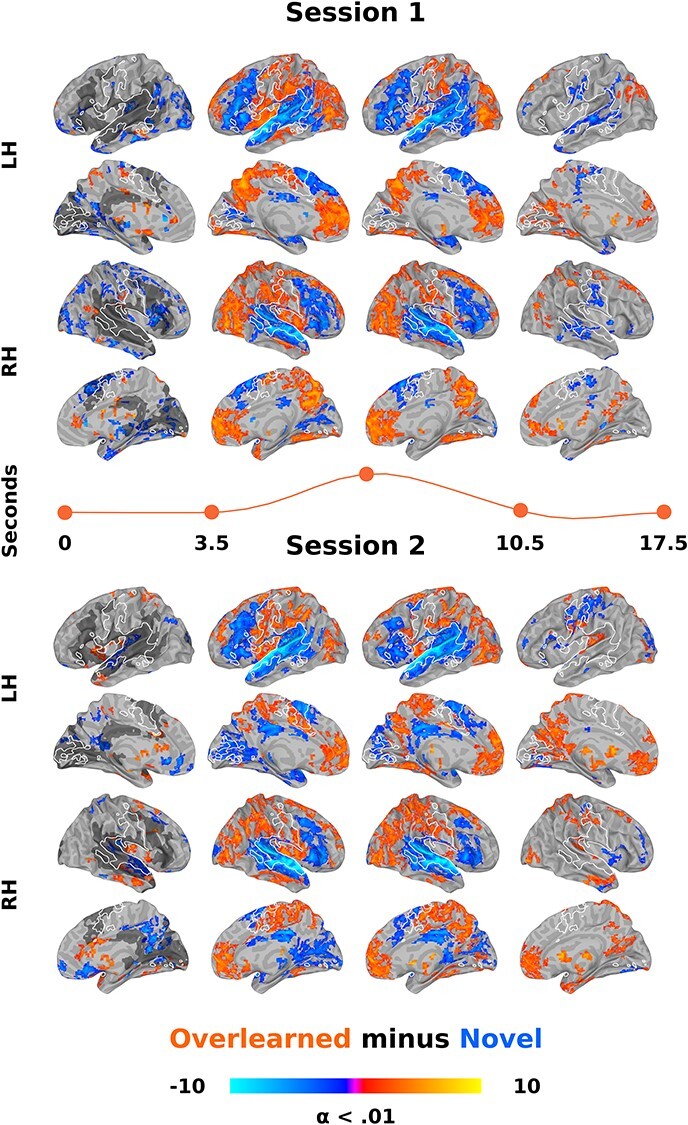
Direct contrast of overlearned minus novel sentence listening for session 1 and 2 across time. GLTs for session 1 (top) and 2 (bottom) for the subtraction of novel from overlearned sentences, done at each of 26 timepoints in the estimated impulse response functions. In both sessions, listening to overlearned sentences results in significantly more activity in sensorimotor regions (reds) also involved in producing speech (white outline). Conversely, overlearned sentences result in less activity in the superior temporal plane and inferior frontal gyrus (blues), specifically in regions that are involved in processing novel sentences (dark gray, left hand column). Everything else is as in Figure 3.

To test whether learning would be specific to overlearned sentences, we conducted a novel sentence listening LMM with session (1 and 2) and timepoint (0–25) as factors. There were no discernible effects of session and no session by time interaction at a cluster size correction for multiple comparisons of alpha (α) < 0.01 (used here and for all subsequent analyses unless otherwise stated). Nonetheless, to assure that there was no effect, we used general linear tests (GLTs) to directly contrast novel sentences for sessions 1 and 2 at all timepoints in a manner used in subsequent analyses (Fig. 3). Over all 26 timepoints, there were few differences from sessions 1 to 2 (Fig. S1). This included 7 clusters with over 20 voxels, with decreases in activity in the cerebellum (*x*/*y*/*z* = 31/−84/−41; 890 voxels), thalamus (*x*/*y*/*z* = −5/−15/7; 42 voxels), and lingual gyrus (*x*/*y*/*z* = −29/−48/−2; 25 voxels) and increases in the left dorsal postcentral gyrus (*x*/*y*/*z* = −41/−39/67; 657 voxels), superior frontal gyrus (*x*/*y*/*z* = −1/32/36; 161 voxels), right superior parietal lobule (*x*/*y*/*z* = 19/−57/70; 99 voxels), and right middle anterior cingulate gyrus (*x*/*y*/*z* = 4/18/25; 20 voxels). We calculated the GLTs for novel sentences for sessions 1 and 2 independently and used these for analysis of the impulse response function (described in the next section; Fig. 4). We also calculated the GLTs for novel sentences, collapsing over sessions 1 and 2 and all 26 timepoints, using this as a guide to language regions (Figs 3 and 5).

#### Overlearning LMM

To test the hypothesis that sentence listening after overlearning involves more sensorimotor and less activity in language regions, an LMM was done with session (1 and 2), sentence (overleaning sentences 1 and 2), and timepoint (0–25) as factors. There were main effects of session and time that encompassed most of the brain at a corrected threshold. In contrast, the main effect of sentence at *P* < 0.01 corrected resulted in 4 clusters in the primary visual cortex (*x*/*y*/*z* = −11/−102/4; 615 voxels), middle occipital cortex around motion area MT+ (*x*/*y*/*z* = −47/−72/7; 123 voxels), superior parietal cortex (*x*/*y*/*z* = 28/−51/61; 121 voxels), and middle frontal gyrus (*x*/*y*/*z* = 43/33/34; 117 voxels). Given this small amount of activity and that these regions are centered around visual cortices, we collapsed over sentences 1 and 2 for all subsequent analyses.

Compared with baseline and independent of time, GLTs for sentences in sessions 1 and 2 both involve processing in the superior temporal plane. However there was an increase in processing in sensorimotor cortices and a large reduction in the spread of activity in the superior temporal plane in session 2 (Fig. S2*A*,*B*). A direct contrast reveals that most of the brain differs at a corrected threshold confirming a bilateral increase in sensorimotor regions and a decrease in the superior temporal plane (Fig. S2*C*). Subcortically, the hippocampus, caudate, and dorsal cerebellum increase, whereas the thalamus, putamen, and ventral cerebellum decrease from sessions 1 to 2. These results are present even at a voxel-wise corrected threshold of *P* < 1 × 10^−10^.

There was an interaction between session and time for overlearned sentences that involved many of these same regions, suggesting that the timing of activity also changes (Fig. 3; Table 2). The previously described results can be seen, with an increase in sensorimotor and a decrease in superior temporal plane regions in session 2. Processing began earlier in sensorimotor regions in session 2 (Fig. 3, first column). This includes activity in primary motor and somatosensory cortex in the central sulcus (*x*/*y*/*z* = −44/−24/55; 376 voxels) and the supplementary motor area (*x*/*y*/*z* = 4/21/49; 40 voxels) and no subcortical structures. Differences in subcortical activity began in the second time bin and are as described above. Using the separately conducted novel sentence listening and production LMMs as a guide, activity for overlearned sentences increases in sensorimotor regions involved in producing speech (Fig. 3, white outline) and decreases in regions active during novel sentence listening (Fig. 3, dark gray).

**Table 2:** Number of voxels (3 mm^3^) in brain regions from the contrast of overlearned sentence listening from sessions 1 to 2 across time as displayed in Figure 3

	Session 1				Session 2			
Region	0–3.5	3.5–7	7–10.5	10.5–17.5+	0–3.5	3.5–7	7–10.5	10.5–17.5+
LH amygdala	0	0	0	0	0	0	0	0
LH angular gyrus	6	17	27	12	0	11	74	0
LH anterior cingulate gyrus and sulcus	0	0	0	1	0	108	149	114
LH anterior occipital sulcus and preoccipital notch	0	6	3	0	0	0	0	0
LH anterior segment of the circular sulcus of the insula	0	0	0	0	0	0	14	26
LH anterior transverse collateral sulcus	0	9	10	16	0	0	0	0
LH calcarine sulcus	33	40	33	18	0	0	4	4
LH caudate	0	24	20	19	0	1	51	32
LH central sulcus	0	0	0	0	79	183	167	165
LH cerebellum	0	819	953	995	0	6	52	68
LH cuneus	69	109	116	50	0	0	7	10
LH frontomarginal gyrus and sulcus	0	0	0	0	0	0	0	0
LH fusiform gyrus	0	31	33	27	0	0	1	0
LH hippocampus	0	5	0	0	0	0	13	8
LH horizontal ramus of the anterior lateral fissure	0	3	0	0	0	0	0	0
LH inferior frontal sulcus	0	0	0	14	0	19	41	43
LH inferior occipital gyrus and sulcus	0	48	88	65	0	0	0	0
LH inferior precentral sulcus	0	0	0	0	0	53	95	79
LH inferior segment of the circular sulcus of the insula	0	20	6	4	0	0	0	0
LH inferior temporal gyrus	0	65	68	49	0	22	49	44
LH inferior temporal sulcus	0	16	14	12	0	0	10	10
LH intraparietal sulcus and transverse parietal sulci	0	3	6	26	0	14	63	3
LH lateral occipito-temporal sulcus	0	9	26	17	0	0	5	1
LH lateral orbital sulcus	0	0	0	0	0	4	6	0
LH lingual gyrus	0	36	36	59	0	10	17	36
LH long insular gyrus and central sulcus of the insula	0	8	10	6	0	0	1	1
LH marginal branch of the cingulate sulcus	0	0	0	0	0	2	1	6
LH medial occipito-temporal and lingual sulci	0	24	33	34	0	0	2	0
LH medial orbital sulcus	0	0	0	0	0	19	29	18
LH middle frontal gyrus	0	0	0	76	0	17	92	53
LH middle frontal sulcus	0	0	0	48	0	0	0	0
LH middle occipital and lunatus sulci	0	7	24	5	0	0	0	0
LH middle occipital gyrus	0	67	137	42	0	0	0	0
LH middle temporal gyrus	0	152	162	113	0	0	0	0
LH middle–anterior cingulate gyrus and sulcus	0	0	9	0	0	5	15	10
LH middle–posterior cingulate gyrus and sulcus	0	0	0	0	0	4	1	7
LH nucleus accumbens	0	0	0	1	0	4	12	17
LH occipital pole	0	16	32	29	0	25	56	60
LH orbital gyrus	0	14	3	3	0	47	88	56
LH orbital sulci	0	0	0	0	0	34	46	39
LH pallidum	0	0	0	27	0	0	0	0
LH paracental gyrus and sulcus	0	0	0	0	34	88	53	27
LH parahippocampal gyrus	0	11	20	11	0	0	21	4
LH parieto-occipital sulcus	13	26	41	6	0	0	0	0
LH pars opercularis	0	31	6	23	0	5	23	14
LH pars orbitalis	0	21	11	9	0	8	8	2
LH pars triangularis	0	2	2	0	0	2	5	5
LH pericallosal sulcus	0	9	8	3	0	0	3	4
LH planum polare	0	52	45	20	0	0	6	8
LH planum temporale	0	76	25	0	0	10	10	0
LH postcentral gyrus	0	0	0	0	69	172	167	162
LH postcentral sulcus	0	0	0	0	23	83	56	50
LH posterior dorsal cingulate gyrus	0	28	15	8	0	2	0	2
LH posterior lateral fissure	0	0	0	0	0	0	0	0
LH posterior transverse collateral sulcus	0	1	7	5	0	0	0	0
LH posterior ventral cingulate gyrus	0	6	7	0	0	0	0	0
LH precentral gyrus	0	0	10	0	13	114	118	104
LH precuneus	13	145	88	72	32	52	19	35
LH putamen	0	79	70	71	0	0	8	6
LH short insular gyri	0	18	19	37	0	0	2	15
LH straight gyrus	0	0	0	0	0	9	13	7
LH subcallosal gyrus	0	0	1	0	0	0	12	18
LH subcentral gyrus and sulcus	0	7	5	3	0	10	16	18
LH suborbital sulcus	0	0	0	0	0	32	47	37
LH subparietal sulcus	0	71	42	46	0	0	0	0
LH sulcus intermedius primus of Jensen	0	1	0	0	0	5	21	0
LH superior frontal gyrus	0	2	0	64	12	370	520	283
LH superior frontal sulcus	0	0	0	0	0	83	107	71
LH superior occipital and transverse occipital sulci	6	11	33	1	0	0	0	0
LH superior parietal lobule	12	47	46	35	171	270	193	117
LH superior precentral sulcus	0	0	0	0	0	14	15	14
LH superior segment of the circular sulcus of the insula	0	4	0	26	0	0	0	0
LH superior temporal gyrus	0	314	266	80	0	0	0	0
LH superior temporal sulcus	0	393	311	134	0	1	29	0
LH superior occipital gyrus	53	72	100	0	0	0	0	0
LH supramarginal gyrus	0	27	1	0	31	137	154	96
LH temporal pole	0	175	193	208	0	0	1	3
LH thalamus	0	2	0	79	0	0	4	5
LH transverse frontopolar gyri and sulci	0	0	0	0	0	0	0	0
LH transverse temporal gyrus	0	5	0	0	0	0	0	0
LH transverse temporal sulcus	0	10	3	0	0	0	0	0
LH ventral diencephalon	0	3	0	8	0	0	5	4
LH vertical ramus of the anterior lateral fissure	0	1	0	4	0	0	0	0
RH amygdala	0	0	12	1	0	1	24	4
RH angular gyrus	0	18	17	4	0	2	0	0
RH anterior cingulate gyrus and sulcus	0	0	0	6	0	97	119	58
RH anterior occipital sulcus and preoccipital notch	0	0	1	1	0	0	0	0
RH anterior segment of the circular sulcus of the insula	0	0	11	0	0	5	20	20
RH anterior transverse collateral sulcus	0	23	16	20	0	5	10	0
RH brainstem	0	139	157	250	0	65	52	6
RH calcarine sulcus	51	65	65	23	0	0	0	0
RH caudate	0	0	3	13	0	57	77	71
RH central sulcus	0	0	0	0	1	63	13	66
RH cerebellum	0	892	1262	1454	0	66	82	17
RH cuneus	71	108	111	43	0	0	4	5
RH frontomarginal gyrus and sulcus	0	1	0	0	0	19	27	32
RH fusiform gyrus	0	87	105	136	0	11	1	0
RH hippocampus	0	13	27	22	0	0	43	0
RH horizontal ramus of the anterior lateral fissure	0	15	20	0	0	0	4	2
RH inferior frontal sulcus	0	0	2	0	0	13	4	14
RH inferior occipital gyrus and sulcus	0	37	51	58	0	7	2	20
RH inferior precentral sulcus	0	0	0	0	0	16	19	33
RH inferior segment of the circular sulcus of the insula	0	5	18	1	0	0	1	0
RH inferior temporal gyrus	0	72	79	74	10	87	17	13
RH inferior temporal sulcus	0	49	25	5	0	7	0	0
RH intraparietal and transverse parietal sulci	0	45	60	47	0	18	1	3
RH lateral occipito-temporal sulcus	0	4	18	11	0	3	2	0
RH lateral orbital sulcus	0	0	0	2	0	4	11	26
RH lingual gyrus	12	60	80	61	0	3	24	15
RH long insular gyrus and central sulcus of the insula	0	0	12	0	0	0	0	0
RH marginal branch of the cingulate sulcus	0	7	0	0	0	0	0	0
RH medial occipito-temporal and lingual sulci	0	52	93	51	0	0	0	0
RH medial orbital sulcus	0	0	0	0	0	25	31	36
RH middle frontal gyrus	0	26	2	29	9	80	49	183
RH middle frontal sulcus	0	4	0	47	0	0	0	0
RH middle occipital gyrus	0	57	64	4	0	0	1	0
RH middle occipital sulcus and lunatus sulcus	0	20	27	0	0	0	0	0
RH middle temporal gyrus	0	223	204	52	1	22	10	14
RH middle–anterior cingulate gyrus and sulcus	0	0	0	0	0	18	18	4
RH middle–posterior cingulate gyrus and sulcus	0	3	3	0	0	24	15	23
RH nucleus accumbens	0	0	0	0	0	13	15	15
RH occipital pole	2	57	64	33	0	17	86	77
RH orbital gyrus	0	17	9	8	0	96	135	150
RH orbital sulci	0	0	0	0	0	44	52	62
RH pallidum	0	0	54	31	0	0	6	10
RH paracental gyrus and sulcus	0	1	0	0	20	94	25	10
RH parahippocampal gyrus	0	20	38	32	0	11	28	5
RH parieto-occipital sulcus	40	61	57	0	0	0	0	0
RH pars opercularis	0	0	24	0	0	11	26	35
RH pars orbitalis	0	29	20	0	0	1	1	7
RH pars triangularis	0	35	34	0	3	60	61	73
RH pericallosal sulcus	0	24	18	5	0	4	3	2
RH planum polare	0	5	5	3	0	24	27	0
RH planum temporale	0	0	0	0	0	0	0	0
RH postcentral gyrus	0	0	0	14	9	74	39	38
RH postcentral sulcus	0	2	7	25	0	37	14	18
RH posterior dorsal cingulate gyrus	0	27	9	0	0	1	0	2
RH posterior lateral fissure	0	0	8	0	0	0	0	0
RH posterior transverse collateral sulcus	0	3	10	6	0	0	0	0
RH posterior ventral cingulate gyrus	0	0	1	0	0	0	0	0
RH precentral gyrus	0	0	0	0	8	59	48	79
RH precuneus	18	218	148	76	17	36	3	4
RH putamen	0	0	102	59	0	13	17	17
RH short insular gyri	0	0	27	2	0	0	10	8
RH straight gyrus	0	0	0	0	0	12	3	2
RH subcallosal gyrus	0	0	0	0	0	23	15	15
RH subcentral gyrus and sulcus	0	0	1	0	0	0	0	0
RH suborbital sulcus	0	0	0	0	0	10	9	8
RH subparietal sulcus	0	66	22	13	0	0	0	0
RH sulcus intermedius primus of Jensen	0	0	0	0	0	0	0	0
RH superior frontal gyrus	0	52	33	82	34	388	357	246
RH superior frontal sulcus	0	23	8	48	0	27	3	56
RH superior occipital and transverse occipital sulci	1	27	52	0	0	0	0	0
RH superior parietal lobule	18	69	77	106	44	107	22	20
RH superior precentral sulcus	0	0	0	0	0	32	25	47
RH superior segment of the circular sulcus of the insula	0	0	55	0	0	0	1	0
RH superior temporal gyrus	0	178	133	2	6	3	27	5
RH superior temporal sulcus	0	287	266	30	0	0	0	0
RH superior occipital gyrus	43	50	64	14	0	0	0	0
RH supramarginal gyrus	0	0	0	0	0	11	2	2
RH temporal pole	0	105	125	166	93	98	28	1
RH thalamus	0	2	35	53	0	0	7	31
RH transverse frontopolar gyri and sulci	0	16	0	31	0	1	1	8
RH transverse temporal gyrus	0	0	0	0	0	0	0	0
RH transverse temporal sulcus	0	5	0	0	0	0	0	0
RH ventral diencephalon	0	0	3	4	0	1	4	0
RH vertical ramus of the anterior lateral fissure	0	3	4	0	0	1	1	4
**Totals**	**461**	**6605**	**7282**	**5979**	**719**	**4082**	**4579**	**3743**

Regions are from the “Destrieux Atlas,” an automatic parcellation used to generate 167 regions (Destrieux et al. 2010).

We further explored overlearning LMM results by visualizing session 1 and 2 timecourses for overlearned sentences. This allows us to better understand if responses for overlearned sentences are a simple redistribution or a reorganization of brain responses. Specifically, we thresholded the GLT contrasting overlearned sentences between sessions 1 and 2 (Fig. S2*C*) at a high *z*-value of 10 and a minimum cluster size of 20 voxels, resulting in 40 clusters. We used this arbitrarily high threshold in order to produce a small number of isolated clusters for display purposes. Nonetheless, so that responses could be discernible in a Figure, we further limited these to 15 clusters, 5 each from 3 sets of regions that correspond to hypothesis, namely the superior temporal plane, frontal/parietal regions, and subcortical structures. The remaining 25 clusters and corresponding time courses are provided in Figure S4.

Overlearned sentence responses in frontal and parietal, including sensorimotor regions, showed a new response in session 2 from below baseline or lack of activity in session 1 (Fig. 4, left). In contrast, superior temporal regions showed a reduction in, lack of or below baseline response in session 2 from a state of heightened activity in session 1 (Fig. 4, middle). Finally, subcortical regions showed an increase in the caudate and a decrease in the cerebellum and putamen for overlearned sentences from sessions 1 to 2 (Fig. 4, right).

#### Overlearning−Novel LMM

To more directly test the hypothesis that sentence processing after overlearning involves more activity in sensorimotor regions and less activity in language regions compared with typical sentence processing, we again performed LMM but after first subtracting the coefficients for novel from overlearned sentences. GLTs for overlearned minus novel sentences in both sessions, independent of time, show that overlearned sentences resulted in greater activity in sensorimotor regions. In contrast, novel sentences produced more activity in the superior temporal plane and inferior frontal gyrus in both sessions (Fig. S3*A*,*B*). Subcortically, in session 1, overlearned sentences produced more brainstem, nucleus accumbens, and dorsal cerebellar activity, whereas novel sentences produced more hippocampal activity. In session 2, overlearned sentences produced more thalamus and more dorsal cerebellum activity, whereas novel sentences produced more ventral cerebellar activity.

**Figure 6:**
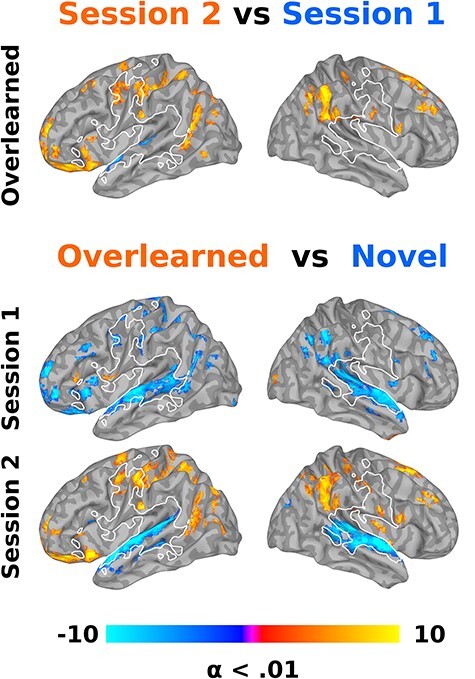
Local connectivity changes after overlearning. A regional homogeneity analysis was conducted to provide a more data-driven validation of more model-based results (shown in Figs 3–5) and to estimate changes in local synchronization or connectivity. In particular, we directly contrasted overlearned sentences between sessions 1 (blues) and 2 (reds; top row). We also contrasted novel (blues) and overlearned sentences (reds) for sessions 1 (middle row) and 2 (bottom row). Results confirm that listening to overlearned sentences after learning results in significantly more local connectivity in sensorimotor regions (reds) and significantly less local connectivity in the superior temporal plane.

There were interactions between session and time for overlearned minus novel sentences that involved much of the brain, though we do not attempt to interpret these here (though Figs 3 and 5 together suggest the direction of these effects). To visualize changes over time as in Figure 3, the GLTs for each time point are presented separately for overlearned minus novel sentences in session 1 (Fig. 5*A*) and session 2 (Fig. 5*B*). The pattern of results clearly shows more sensorimotor activity for overlearned sentences in both sessions (Fig. 5*A*,*B*, reds). The speech production LMM shows that this occurs in similar sensorimotor regions used to produce novel sentences. Conversely, there was less superior temporal plane and inferior frontal gyrus activity for overlearned sentences (Fig. 5*A*,*B*, blues). These regions closely overlapped those for novel sentences (Fig. 5, dark gray).

#### Regional Homogeneity

The deconvolution and subsequent LMM results are model-based, aggregating over stimuli, though without a priori assumptions about the shape of the hemodynamic response. To test whether there is support for hypotheses in a more model-free manner and whether there is more local sensorimotor connectivity after overlearning, we conducted a regional homogeneity analysis on preprocessed time series. There was an increase in sensorimotor cortices and a decrease in superior temporal plane local connectivity from sessions 1 to 2 for overlearned sentences (among other regions; Fig. 6, top). We then contrasted overlearned and novel sentences for sessions 1 and 2 separately (Fig. 6, bottom). In session 1, there were few regions more active for overlearned sentences, whereas novel sentences resulted in significantly greater regional connectivity, mostly throughout the superior temporal plane and inferior parietal regions, bilaterally. In contrast, the overlearned sentences produced greater inferior parietal and bilateral sensorimotor local connectivity in session 2 and the local superior temporal plane connectivity remained greater for novel sentences (Fig. 6, bottom).

#### Network

**Figure 7:**
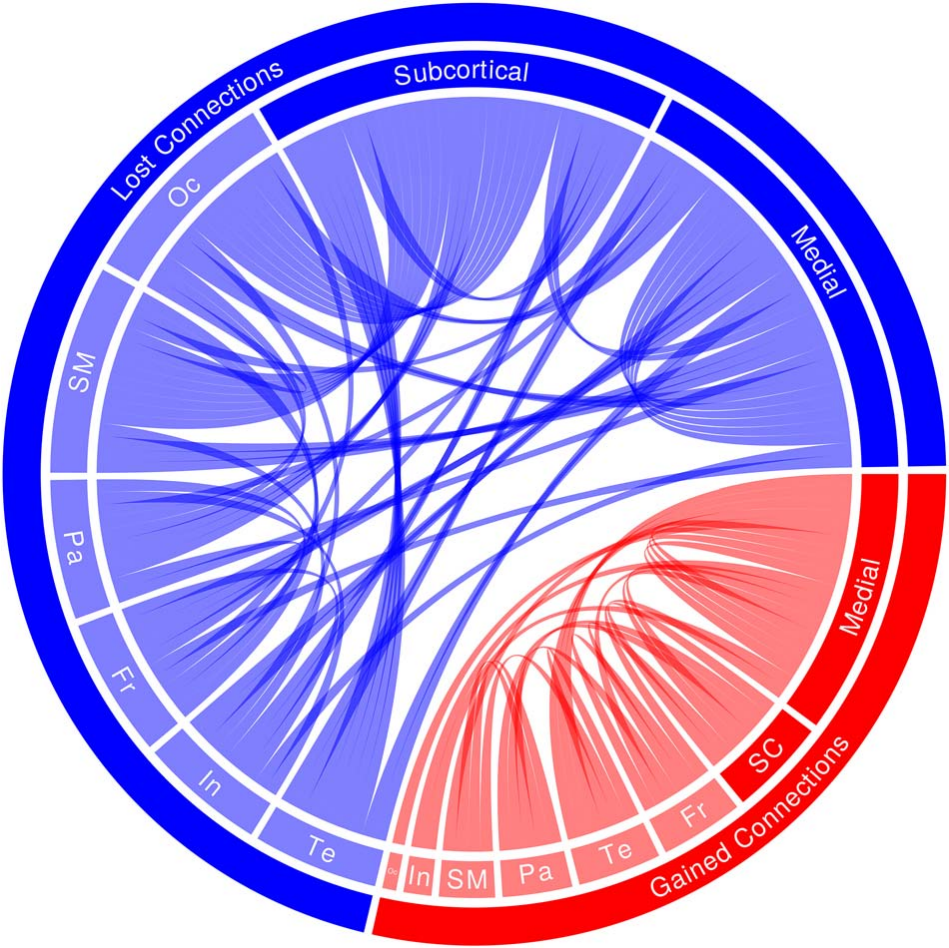
Changes in network connectivity after overlearning. The chord plot shows all 90 lost (blue) and gained (red) connections between sessions 1 and 2 as determined by chi-square tests on binarised connections between pairs of 167 regions of interest (𝒳^2^ > 6.63; *P* < 0.01). For ease of visualization, these were grouped into frontal (Fr), insula (In), medial, occipital (Oc), parietal (Pa), sensorimotor (SM), subcortical (SC), and temporal (Te) regions. Results suggest that sentence overlearning results in significantly less connectivity, particularly in medial cortical and subcortical regions.

To further test the hypothesis that the brain reorganizes after overlearning, we analyzed the global network variation between session 1 and 2 for overlearned sentences using edit distance. There was significant change in connectivity, averaging 45.4% change (SD = 5.50%; Minimum = 32.26%; Maximum = 55.37%; *t*_11_ = 28.69, *P* = 1.08 × 10^−11^). To visualize some of these changes in connectivity, we did chi-square (𝒳^2^) tests on the binarised connections, using a threshold of 6.63 (*P* < 0.01; Fig. 7). This resulted in 90 changes in connections, with 25 connections gained (27.77%) and 65 connections lost (72.22%). This large reduction in connections was across all major brain subdivisions. Nonetheless, 80% of the changes included medial/midline (42) and/or subcortical structures (34, with 4 overlapping medial–subcortical connections). Of the 42 medial regions, 27 were lost connections (64.29%). Of the 34 subcortical regions, 28 were lost connections (82.35%). New subcortical connections involved the amygdala (×1), cerebellum (×1x), pallidum (×1x), hippocampus (×2x), and nucleus accumbens (×2x). Lost subcortical connectivity involved the caudate (×1x), amygdala (×2x), cerebellum (×2x), hippocampus (×2x), pallidum (×2x), brainstem (×4x), putamen (5x), ventral diencephalon (×6x), and thalamus (×6x). At a more stringent threshold (*P* < 0.005), the disproportionate number of lost connections remains similar at 75.93%, containing more medial and subcortical lost connections (85.19%; Table S1 for more information about changes in connectivity).

To characterize global connectivity changes with a more robust method, Deltacon similarity was computed. There was a significant dissimilarity between sessions with an average 46.1% change (SD = 3.90%; Minimum = 43.20%; Maximum = 52.33%; *t*_11_ = 41.43, *P* = 1.97 × 10^−13^). To further understand network changes, we explored a number of other global and local measures. None of density, diffusion, efficiency, or flow were significantly different from sessions 1 to 2 (*P*s > 0.05). There were also no differences in the number of communities or the measures of centrality (*P*s > 0.05).

## Discussion

Based on the relative preservation of both sensorimotor regions and formulaic language in aphasia and Alzheimer’s disease (Van Lancker Sidtis and Sidtis 2018) and proposed predictive models of the role of speech production related regions in speech perception (Skipper et al. 2017), we hypothesized that sentences would be processed by sensorimotor regions and not language regions after learning in our paradigm (Fig. 1). Suggesting overlearning occurred, reaction times to complete the final word of overlearned sentences decreased by 2.1 s (Fig. 2*A*), identification of other words from those sentences was faster (Fig. 2*B*), and the sentences were remembered with higher accuracy in session 2 (Fig. 2*C*).

Overlearning corresponded to a considerable and local increase in sensorimotor region activity around the central sulcus, including primary motor and somatosensory cortices in both model-based and model-free analyses (Figs 3,5, and6, oranges). These regions overlapped with those involved in producing novel sentences (Figs 3,5, and6, white outline). After overlearning, speech perception begins earlier in sensorimotor regions, before the hemodynamic response is typically expected to rise (Figs 3 and 5, left) and before superior temporal regions (compare Fig. 4, left and middle onsets). Consistent with reorganization, some of these regions had a hemodynamic response around zero before overlearning (Fig. 4, left).

Concomitantly, we found a dramatic bilateral reduction in activity and local processing in the superior temporal plane when comparing overlearned sentences from sessions 1 to 2 (Fig. 3, blues; Fig. 6, blues, top). When comparing to novel sentences, there was a reduction in activity and local processing in superior temporal plane and the inferior frontal gyrus language regions (Fig. 5, blues and gray; Fig. 6, blues, bottom). Consistent with reorganization, some superior temporal plane regions had a hemodynamic response around zero after overlearning, suggesting they were not active on average (Fig. 4, middle).

Global measures of network variation indicate that about 45% of the brain reorganized after overlearning, with few new connections and a profound decrease in connections across the whole brain (Fig. 7). The latter mainly involved medial cortical and subcortical brain regions. Lost subcortical connections involved the amygdala, basal ganglia (mostly the putamen but also the caudate), brainstem, cerebellum, hippocampus, thalamus, and ventral diencephalon. These subcortical results parallel those in voxel-based analyses that showed less increases and more decreases in activity (e.g., Fig. 4, right). Other local and global network measures were not significant. With regard to the local measures, perhaps central regions did not change, whereas their connectivity did and community numbers may have remained similar, whereas the communities themselves changed dramatically. With regard to the lack of effect for the other global measures, perhaps these are robust to local connectivity variations between sessions. For example, global efficiency is likely to remain stable in both structural and functional networks over time even while local efficiency significantly changes (Dennis et al. 2013; Cao et al. 2014).

Overall, results suggest that the brain mechanisms associated with perceiving overlearned speech are qualitatively different from less formulaic or more compositional language. They suggest that when speech segments become sufficiently overlearned, they are processed by a much more circumscribed and cortically isolated set of sensorimotor regions involved in producing speech and little, if at all, by language regions.

### Formulaic Production

It has been proposed that formulaic language production is supported by right hemisphere and subcortical interactions (Van Lancker Sidtis and Sidtis 2018). This conclusion was based on research showing that formulaic expressions are more common in left compared with right hemispheric damage (though see Baldo et al. 2016; Zimmerer et al. 2018). It was additionally based on results suggesting that individuals with Alzheimer’s disease produce more formulaic language than people with basal ganglia strokes and Parkinson’s disease. This is attributed to relative preservation of the basal ganglia in Alzheimer’s disease. A region of interest-based neuroimaging study in healthy people supports both arguments, showing that increased formulaic language production is correlated with increased right inferior frontal gyrus and decreased left caudate activity (Sidtis et al. 2018).

Like Sidtis et al. (2018), we also show a large increase in right inferior frontal gyrus and decrease in subcortical activity (Fig. 3, bottom). However, our results in other regions are more bilateral and centered around sensorimotor rather than subcortical regions. This might suggest that the postulated right hemisphere locus is due to a stronger weighting of right hemisphere regions after aphasia. Our results also suggest that the presumed subcortical locus might be less about the preservation or deterioration of the basal ganglia in Alzheimer’s and Parkinson’s disease and more about preserved sensorimotor regions in the former. This is more consistent with research suggesting that the basal ganglia is significantly impacted in Alzheimer’s disease, even in early stages (Cho et al. 2014; Pini et al. 2016; Tentolouris-Piperas et al. 2017). Collectively, our results suggest that the more preserved right sensorimotor and not right language regions or subcortical structures are the locus of formulaic speech production in aphasia and Alzheimer’s disease.

### Formulaic Comprehension

Our results are more consistent with neuroimaging studies of formulaic language comprehension. Much of this work centers around figurative language, like idioms and metaphors. These are processed bilaterally in language regions, with varying contribution of the left and right hemispheres as a function of familiarity (Mashal et al. 2008; Hillert and Buračas 2009; Kasparian 2013; Yang 2014). Furthermore, the more familiar (Schmidt and Seger 2009; Cardillo et al. 2012), frequent (Fiez et al. 1999; Blumenthal-Dramé et al. 2017), or coherent multiword expressions are (Bhattasali et al. 2018, 2019), the less language regions tend to be active. Some studies show that sensorimotor activity is more strongly associated with high-frequency words, more coherent word composition, and impairments of word composition (Bemis and Pylkkänen 2011; Price et al. 2015), whereas the basal ganglia are less active for higher frequency words (Graves et al. 2010; Blumenthal-Dramé et al. 2017). One study concluded that “areas canonically implicated in traditional neurophysiological models of language processing appear to play a lesser role in basic [more coherent] composition” (p. 2802) (Bemis and Pylkkänen 2011). Another concluded that multiword expressions rely on regions other than “traditional frontal and temporal nodes of the language network” (p. 12) (Bhattasali et al. 2018).

### Sequence Learning

The strongest similarities to our results derive from motor sequence learning research. This work shows that becoming or being an expert motor performer involves a well-documented set of decreases and increases in brain activity that depend on the length of learning (Ashby et al. 2010; Lohse et al. 2014; Doyon et al. 2018; Caligiore et al. 2019). Specifically, there are 2 learning stages associated with relative duration: fast online learning, described as more explicit and by repetition suppression and slow learning, described as more implicit and by repetition enhancement and sleep-related or offline consolidation. Fast learning is frequently linked to more associative cortical and subcortical regions. In contrast, slow learning is associated with a global decrease in activity in most regions, including prefrontal, premotor, parietal, sensorimotor, and subcortical structures like more associative basal ganglia and cerebellar regions. Among these widespread decreases, there is a selective increase in some sensorimotor and subcortical regions, including less associative aspects of the basal ganglia and cerebellum. Generally, these changes might be described as a shift away from cognitive systems (involving attentional, inhibition, control, etc.) and toward more “automatic” sensorimotor brain regions.

Our study incorporated early and late learning phases, with an early online perceptual learning period (measured in fMRI session 1) and a late offline production learning period with motor memory consolidation (measured in fMRI session 2). Consistent with slow learning, we show global decreases in activity in most cortical and subcortical regions with a selective focal increase in some sensorimotor and subcortical regions. Also consistent with the distinction between fast and slow learning, our results do not simply constitute a redistribution of activity patterns in the same language regions but, rather, a reorganization to sensorimotor regions, suggesting different processes underlying the perception of novel and overlearned sentences (Kelly and Garavan 2005).

### Neurobiological Models

More generally, our results suggest a model by which the perception of overlearned speech may not rely on language regions. Like test–retest reliability and lexical processing studies, our results suggest that the neurobiology of speech perception and language comprehension is more variable and distributed than posited by classical and contemporary models (Hickok and Poeppel 2007; Friederici and Gierhan 2013; Fedorenko and Thompson-Schill 2014; Tremblay and Dick 2016).

If so, an open question becomes: What differentiates the more distributed regions found in some studies from the static regions in popular models? One hypothesis is that the whole brain variously participates in speech perception and language comprehension and that language regions as we know them are mostly connectivity hubs coordinating this more distributed system (Skipper 2015). Because of the reliance on measures of central tendency, these distributed regions are “averaged out” in most studies (Skipper 2015). They only become obvious when specific categories are under examination, for example, individual differences, action words, or, as here, formulaic language.

If they are mostly hubs, the focus on language regions or their “homologues” in therapeutic interventions risk overemphasizing the importance of less specific regions and neglecting more behaviorally relevant network nodes. For example, given the preservation of formulaic language and corresponding sensorimotor regions in some individuals with aphasia and Alzheimer’s disease, it makes sense to focus on those expressions and regions as they might be used as a scaffold for language recovery. Indeed, use of formulaic song and language shows promise in therapy (Stahl and Van Lancker Sidtis 2015; Stahl et al. 2020).

To the extent models guide therapy, this proposal requires us to move beyond current neurobiological models with static regions (Skipper 2015; Skipper et al. 2017; Hula et al. 2020; Upton and Hope 2020). It begs for a more detailed neurobiological account of overlearned expressions and other factors that result in differently distributed language processes. It also suggests a greater focus on item analysis and individual differences and a reduced reliance on measures of central tendency to understand the organization of language and the brain (Seghier and Price 2018).

### Limitations

There are a number of possible methodical limitations to this work associated with the behavioral and fMRI tasks and interpretations. With respect to the behavioral methods, though all 3 tasks showed effects consistent with overlearning, the lexical decision task was likely underpowered with only 21 items. The sentence recall task might have used spoken responses given this is how participants trained. Finally, our procedure might not reflect natural overlearning and formulaic language. As in our study, overlearning does often occur through repetition in short sessions over a relatively small period of time (e.g., as in learning at a university over weeks for quizzes and exams). However, the overlearning associated with formulaic language is more likely picked up over a lifetime of repetitions. Similarly, though many overlearned expressions are not particularly meaningful (e.g., “whats up”), numerous have more specific meanings. Our repetition learning task did not unfold over years nor did it emphasize semantic content per se. Thus, the neurobiology of more extensive overlearning, with more semantically meaningful content might differ somewhat from what was observed here.

In terms of fMRI methods limitations, a larger sample size would have been preferable. Nonetheless, the high number of stimulus repetitions, optimized stimulus presentation design, high sampling rate, and within-participant design largely mitigate this concern (Zandbelt et al. 2008; Bennett and Miller 2010). Next, our design likely leads to repetition suppression effects for the overlearned but not the novel sentences because the former are repeated (Grill-Spector et al. 2006). This does not affect interpretation of the main body of results because these involve direct comparisons between overlearned sentences from sessions 1 to 2 where repetition effects are equivalent (Figs 3–5 upper panel and Fig. 7). However, it might temper interpretations of follow-up analyses involving both the novel and overlearned sentences (Figs 5 and 6 lower panel). Alternately, these results are even more impressive given that sensorimotor regions are more active for overlearned sentences despite repetition suppression.

Another limitation is that we used a production task that was only based on novel sentences. Though our study was about speech perception, having an additional production task with overlearned sentences would have allowed us to also make conclusions about the production of overlearned sentences, making more contact with some of the work that inspired our study. Though our novel sentence production task was used as a guide only, it could be that the production of overlearned sentences includes more dorsal sensorimotor regions, presupplementary motor area, and prefrontal cortex and less supplementary motor area as was observed for listening to overlearned sentences.

Finally, the passive design did not permit us to collect behavioral data in the scanner to directly correlate with activity patterns. We felt this was justified because our hypotheses centered around sensorimotor systems. Had we included a motor response, there would be no way to exclude contamination of results from associated movements from sensorimotor activation. We take solace that the behavioral effect sizes we observe outside of the scanner were unusually large, decreasing the likelihood of alternative explanations.

## Conclusions

Results suggest that the brain regions supporting speech perception are not fixed but, rather, dramatically reorganize as a function of individual experience with speech production. Specifically, repeated experience speaking the same word sequence seems to change the memory representation of those words to be more formulaic. This trace is subsequently used by the brain in the process of speech perception, perhaps in a predictive manner. This involves a different set of regions than is said to support more compositional language. Given how frequently formulaic expressions occur, how fast they are processed, and how important they are in learning, results call for more research on how overlearning occurs and formulaic speech is processed. They also suggest why formulaic expressions might be preserved in aphasia and Alzheimer’s disease. Therapy for such disorders is often informed by the belief that language is supported by a static set of language regions or the language network, formalized in classical and contemporary neurobiological models. Given that this is not the case, as shown here and elsewhere, future interventions might benefit from adopting more dynamic and distributed network models of language and the brain (Skipper 2015).

## Supplementary Material

Supplementary material can be found at *Cerebral Cortex* online.

## Author Contributions

D.R.L. and J.I.S. conceived of the study. S.B., D.R.L., E.M., and J.I.S. designed the study. All authors helped with stimulus pretesting and E.M. selected overlearned sentences. J.I.S. wrote the manuscript with help from S.A., S.B., Y.J.J., and D.R.L. E.M., S.B., S.L., and Y.J.J. collected the data. D.R.L. did behavioral analyses. S.L. and Y.J.J. did the ICA artifact labelling for fMRI preprocessing, checked by J.I.S. J.I.S. did all fMRI preprocessing and analyses except the network analyses, done by S.A.

## Supplementary Material

Skipper_et_al_Sentence_Overlearning_Supplementary_Material_bhab354

## Notes

J.I.S. would like to thank the fine people at the Birkbeck-UCL Centre for Neuroimaging (BUCNI). He also thanks Marple, Prunella, Rupert, and BB for their support. *Conflict of Interest:* None declared.
